# How should spontaneous breathing trials be performed in the light of recent literature?

**DOI:** 10.1186/s13613-025-01507-2

**Published:** 2025-07-16

**Authors:** Arnaud W. Thille, Gonzalo Hernández

**Affiliations:** 1 Service de Médecine Intensive Réanimation, CHU de Poitiers, 2 rue la Milétrie, Poitiers CEDEX, 86021 France; 2 CIC 14-02, IS-ALIVE research group, University of Poitiers, Poitiers, France; 3 Complejo Hospitalario Universitario de Toledo, Toledo, Spain; 4 Ciber Enfermedades Respiratorias (CIBERES), Instituto de Salud Carlos III, Madrid, Spain; 5 Grupo de Investigación en Disfunción y Fallo Orgánico en la Agresión, Instituto de Investigación Hospital Universitario la Paz (IdiPAZ), Madrid, Spain; 6 Universidad Alfonso X el Sabio, Madrid, Spain

Dear Editor,

The decision of extubation is difficult in intensive care units (ICUs) because mortality is particularly high in case of reintubation. Reintubation rates are around 10–15% but may exceed 20% in patients at high risk of extubation failure, i.e. patients older than 65y, with any underlying cardiac or respiratory disease, or with prolonged duration of mechanical ventilation. To reduce that risk, guidelines recommend to systematically performing a spontaneous-breathing trial (SBT) before extubation of all patients mechanically ventilated for at least 24 h in an ICU, with the aim at replicating postextubation physiological conditions. SBTs are most commonly performed either with low levels of pressure-support ventilation (PSV), or with a T-piece by simply disconnecting the patient from the ventilator and providing additional oxygen. Whereas SBTs performed with a T-piece accurately replicate post-extubation physiologic conditions, SBTs performed with PSV are less challenging in terms of work of breathing [1]. Low levels of PSV were initially justified to reduce the work imposed by the ventilator circuit/valve and endotracheal tube. However, due to technological improvements of ICU ventilators, especially the use of high-technology regulated servo-valves maintaining continuous flow-by as well high sensitivity of inspiratory trigger, the work load has decreased considerably, and SBTs with PSV significantly underestimate work of breathing needed after extubation. Despite that, clinical trials have never shown an increased risk of reintubation using SBTs with PSV. As compared to SBTs with a T-piece, SBTs with PSV are easier to pass and a higher proportion of patients may be extubated promptly after the initial SBT [2, 3]. A clinical trial has compared SBTs performed with PSV (8 cm H_2_O) and no positive end-expiratory pressure (PEEP) for only 30 min or with a T-piece for 2 h in 1153 patients intubated for at least 24 h [2]. The proportion of patients extubated after the first SBT and not reintubated within the 72 h following extubation was significantly higher with PSV than with a T-piece. Indeed, patients were more likely to be extubated after the first SBT when performed with PSV than when performed with a T-piece, while reintubation rates were exactly the same. However, this study included a majority of patients at low risk of extubation failure, and these findings might not be extrapolable to all ICU patients. Another clinical trial including 969 patients at high risk of extubation failure (age > 65 years, or with any underlying cardiac or respiratory disease) compared SBTs performed with PSV (8 cm H_2_O) and no PEEP or with a T-piece for around one hour [3]. Although the number of ventilator-free days at day 28 was similar between groups, the proportion of patients extubated within 24 h following the first SBT was again significantly higher with PSV than with a T-piece, while reintubation rates did not significantly differ between groups. Therefore, two recent large-scale randomized controlled trials show that SBTs with PSV and no PEEP may hasten extubation decision-making without increased risk of reintubation, even in patients at high risk of extubation failure [2, 3]. Additionally, SBTs with PSV are easier to perform than a T-piece since they involve no ventilator disconnection, require no additional materials, and especially no disposable material, that is consequently cheaper and more eco-friendly, and allow continuous monitoring of tidal volumes and respiratory rate on the ventilator screen. Therefore, SBTs with PSV should be preferred as a first option assessing extubation readiness in ICUs.

Another question is whether or not PEEP should be added during SBTs. Addition of PEEP (usually 5 cm H_2_O) may further decrease work of breathing and may further hasten extubation as compared with no PEEP. However, it could render the SBT too easy to pass, thereby increasing the risk of post-extubation respiratory failure. In a recent multicenter clinical trial, SBT performed with PSV 8 cm H_2_O and PEEP 5 cm H_2_O reduced time to extubation compared to SBT with PSV (5 cm H_2_O) and no PEEP, without increased risk of reintubation [4]. However, this study included a majority of patients at low risk of extubation failure, and the results could not be extrapolable to those at high risk of extubation failure. In a single-center study recently published in *Annals of Intensive Care* including 98 patients having failed a first SBT, risk of reintubation was again not different between SBTs with PSV (7 cm H_2_O) and PEEP (5 cm H_2_O) or with a T-piece [5]. Although SBT performed with PSV and additional PEEP is less challenging, it does not seem associated with increased risk of reintubation. However, further studies are needed to generalize such an easy trial in daily practice. Indeed, recent findings should be interpreted with caution since no large-scale clinical trial has compared SBTs with PSV and additional PEEP vs. a more challenging SBT with PSV and no PEEP or with a T-piece in patients at high risk of extubation failure. It should be noted that even if SBT with a T-piece is a more challenging test, accurately replicating the work of breathing after extubation, it has not been shown to lead to a reduced risk of reintubation. In fact, reintubation is often multifactorial and may be influenced by noninvasive respiratory supports initiated after extubation. Prophylactic noninvasive ventilation significantly decreases the risk of reintubation in patients at high risk of extubation failure, and probably has more impact that the challenge represented by SBTs. Whether a more challenging SBT performed with a T-piece is useful in the highly specific population of patients with neuromuscular diseases such as myasthenia gravis or Guillain-Barré syndrome, which are characterized by respiratory muscle fatigue, need further exploration.

In conclusion, a more challenging SBT with a T-piece fails to reduce risk of reintubation. In contrast, a less challenging SBT with PSV and no PEEP is easier to pass than with a T-piece, and may hasten extubation without increased risk of reintubation. According to recent literature, SBT with PSV and no PEEP should be the first option assessing extubation readiness in ICUs. Whether an even less challenging SBT with PSV and PEEP is safe requires further research.

## Data Availability

The manuscript has no associated data.
